# Transactions of the Medical Association of the State of Alabama

**Published:** 1876-09

**Authors:** 


Transactions of the Medical Association of the State of Alabama.—We have received the volume of Transactions of the Alabama Medical Association and State Board of Health for the session of 1876. We find in this volume of 270 pages but two papers: the first a "Prize Essay on the Pathology and Treatment of Bright's Disease." By Dr. H. D. Schmidt, of Mobile, Alabama. This is a well-written and exhaustive paper of 80 pages, in which is discussed some new features, or rather some of its pathological features are discussed in connection with similar pathological conditions of other diseases, from which valuable deductions are arrived at. The following extract will give the object and scope of the essay:

"In presenting the following pages to the profession, it is not with a view of adding some newly-discovered facts to the pathology of Bright's Disease, a disorder which, ever since it was first described by the author whose name it bears, has been very thoroughly investigated by the most eminent pathologists of our time. The object in view is rather to review and trace back to their origin the various phenomena observed in its course, which, taken together, constitute it; and farther, to compare the organic changes occurring in the tissues of certain organs, especially the kidneys, in the different stages of this disease, with similar changes which I have observed to take place in the same organs in fatal cases of another grave affection, namely, *Yellow Fever.*"

The second paper is by Dr. R. D. Webb, of Livingston, Alabama, on "Hæmorrhagic Malarial Fever." In this paper of 107 pages, Dr. Webb gives us the literature of the affection as it has appeared under different names in different countrys and localities, and then discusses the disease and its peculiarities as it has appeared in the Southern States within the past ten years. We regard it a most excellent paper. The Publishing Committee, in the following card, tell us that valuable papers have been omitted:

"The Committee on Publication deeply regret that the financial condition of the Association for the year 1876 would not permit them to publish a larger volume than the one they now present to the medical public. They are compelled to omit *fifteen* valuable papers which reflect much credit on the Association, as well as on their authors.

We have again intrusted the printing to Messrs. Barrett & Brown, of Montgomery, and feel satisfied that these gentlemen will meet all expectations.

"Respectfully submitted.

B. H. Riggs,
J. B. Gaston,
R. F. Michel,
*Publishing Committee.*"

For the same reason all the papers presented to the Medical Association of Georgia in 1875 were omitted, and the present year many valuable papers were not published for the same reason. This is all wrong, and certainly very unfortunate, and will soon seriously impair the usefulness of the Association, by reducing its laborers, as no one is disposed to give the time and labor necessary to perfect a paper upon any subject, when the probabilities are that it is to be buried in the manuscript archives of the Association, without the possibility, even in the distant future, of its ever being brought to light.

